# Morphology of the Physiological Foramen: A Systematic Review

**DOI:** 10.3390/dj13120581

**Published:** 2025-12-05

**Authors:** Thomas Gerhard Wolf, Samuel Basmaci, Sophia Magdalena Weiberlenn, David Donnermeyer, Andrea Lisa Waber

**Affiliations:** 1 Department of Restorative, Preventive and Pediatric Dentistry, School of Dental Medicine, University of Bern, 3010 Bern, Switzerland; 2 Department of Periodontology and Operative Dentistry, University Medical Center, Johannes Gutenberg University Mainz, 55131 Mainz, Germany

**Keywords:** anatomic landmarks, apical constriction, diameter, foramina, tooth apex

## Abstract

**Objective**: Accurate knowledge of apical morphology is crucial for determining the correct working length and achieving an optimal seal, both of which are vital for long-term endodontic success. This review summarizes and evaluates the current literature on the physiological foramen, focusing on its diameter and the distance between the anatomical apex and the physiological foramen. **Materials and Methods**: A systematic literature search was conducted using the databases PubMed (via Medline), Embase, LILACS, and Scopus. Studies addressing the anatomy of the physiological foramen were selected based on predefined inclusion criteria. A total of 743 records were identified. After removing 103 duplicates, the titles and abstract of 640 records were screened, with 625 being excluded as irrelevant. Fifteen full texts were assessed and six excluded for not meeting inclusion criteria. Five additional articles were found through manual search. In total, 14 studies were included in the review. The risk of bias was assessed using the AQUA tool. **Results**: Considerable variation in the diameter of the physiological foramen was observed across the included studies, ranging from 0.15 mm to 0.43 mm depending on tooth type and location. Additionally, the distance between the anatomical apex and the physiological foramen varied from 0.1 mm to 1.2 mm. **Conclusions**: The results demonstrate considerable heterogeneity in the dimensions and position of the physiological foramen, with oval shapes occurring more frequently than round or irregular ones. Standardized definitions and consistent terminology are essential to improve comparability across studies and to enhance the clinical applicability of research findings. Recognizing these anatomical variations optimizes endodontic treatment outcomes and minimizes procedural errors.

## 1. Introduction

Successful endodontic treatment requires precise preparation and complete obturation of the root canal system. Incomplete sealing is one of the most common causes of endodontic failure [1,2]. The radiographic foramen represents the outermost apical point of the root apex of the tooth, which is visible on a radiograph but does not necessarily correspond to the actual root apex. In contrast, the anatomical foramen describes the outermost area of the apex of a tooth where the pulp merges into the surrounding tissue, regardless of the radiographic representation. The physiological foramen, also known as the apical constriction, is the narrowest part of the root canal in the apical region and is often considered the apical limit for root canal treatment [3,4]. Kuttler originally defined the physiological foramen at the cement–dentin junction [5], but studies show that there is considerable variation in shape and location, including parallel and nonconical apical constrictions [1,2,3,4]. The term “cement–dentin junction”, which is often confused with “apical constriction”, is anatomically misleading because the cement–dentin junction is not only found in the apical region but along the entire root. Hülsmann & Schäfer emphasize that complete removal of the infected tissue is the main goal of root canal treatment [6]. Instrumentation should therefore be performed up to the physiological foramen without exceeding it to preserve the minimal wound area between the pulp and periapical tissue [7,8,9]. Excessive application of the root filling material beyond the physiological foramen may therefore jeopardize clinical success [7,8,9]. The shape and diameter of the physiologic foramen are crucial for planning and performing the preparation of the apical third [7,10]. The aim of this systematic review was to identify studies on the morphology of the apex and to obtain information that would enable the clinician to make better treatment decisions and to provide insights into the understanding of the different structures.

## 2. Materials and Methods

A comprehensive literature search was conducted to investigate the anatomy, physiology, and clinical relevance of the physiological foramen in dentistry. The databases PubMed (via MEDLINE), Embase, LILACS, and Scopus were searched up to July 2025. This review was reported in accordance with the Preferred Reporting Items for Systematic Reviews and Meta-Analyses (PRISMA) [11]. The corresponding PRISMA checklist is presented in the Supplementary File (Supplementary Material File S1. PRISMA checklist). The study protocol was registered in the International Prospective Register of Systematic Reviews (PROSPERO) under ID CRD420251138377.

### 2.1. Selection Criteria

To identify relevant publications, a search string was developed based on predefined inclusion criteria. Studies were eligible if they examined the anatomical characteristics, physiological function, or endodontic significance of the physiological foramen. The search strategy included the following MeSH terms:

((pulp chamber floor) OR (anatomical apex) OR (apical portal) OR (exit of the apical portal) OR (opening of the root canal) OR (endodontic apex) OR (apical delta) OR (apex) OR (radiographic apex) OR (apical delta of the root canal)) AND ((apical constriction of the root) OR (physiological foramen geometry) OR (morphology of the physiological apical foramen) OR (physiologic apex) OR (radiographic terminus of the root canal) OR (apical region of the root canal)) AND ((physiological foramen) OR (apical foramen) OR (cemento-dental junction) OR (apical anatomy) OR (radiographic terminus of the canal) OR (endodontic main foramen) OR (opening of the root canal))

No language or publication date restrictions were applied. The search results were screened manually to ensure relevance to the research question. Duplicate studies were removed, and the remaining articles were first screened by two independent reviewers according to title and abstract (S.B. and A.L.W.). Disagreements were resolved by consensus or by consulting a third reviewer (T.G.W.). The relevant articles were subjected to a full-text review by the same reviewers. Studies were included if they focused on the morphology or spatial position of the physiological foramen in relation to anatomical or radiographic landmarks. Review articles, case reports, and studies not directly addressing the physiological foramen were excluded.

### 2.2. Risk of Bias Assessment

The AQUA tool (Anatomic Quality Assessment) [12] was applied to assess the quality and risk of bias of the included studies (S.B. and A.L.W.). This evaluation considered domains such as study design, sample selection, methodology transparency, and reproducibility of measurements. Each study was categorized as having low, moderate, or high risk of bias. Disagreements in the assessment were resolved by consensus or through a third independent reviewer (T.G.W.).

### 2.3. Data Extraction

Relevant data including authorship, publication year, sample size, tooth type, methods of measurement, and main findings were extracted and tabulated. Descriptive synthesis of results was used due to heterogeneity in study designs and outcomes. No meta-analysis was performed.

## 3. Results

**Figure 1:**
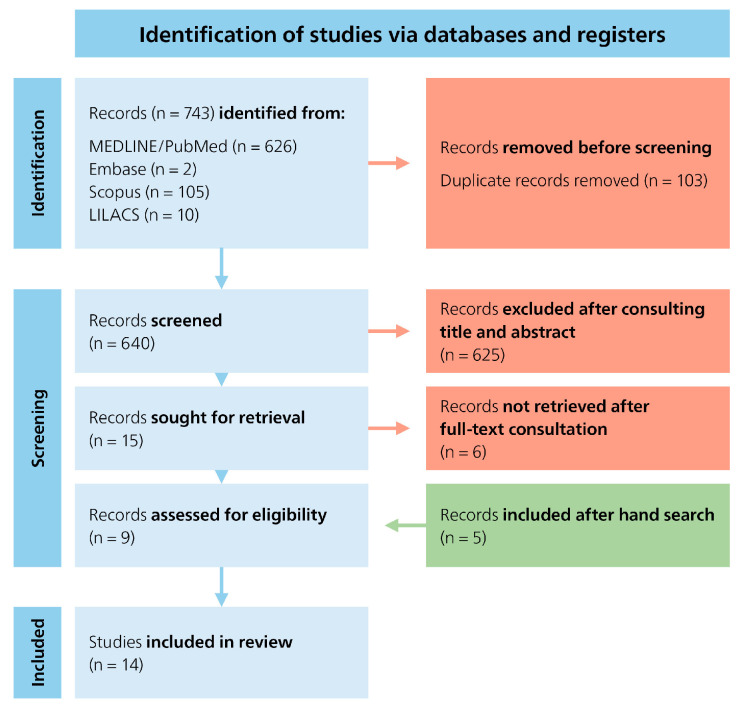
PRISMA flowchart.

A systematic literature search was conducted across four electronic databases (PubMed (via Medline), Embase, Scopus, and LILACS)) and initially yielded a total of 743 articles. After removal of 103 duplicates, 640 records remained and were screened based on title and abstract. Of these, 625 records were excluded due to irrelevance to the research question. The full texts of the remaining 15 articles were assessed in detail. Following this full-text review, 6 articles were excluded for not meeting the inclusion criteria. 5 additional articles were identified through manual searching of reference lists. Ultimately, 14 studies were included in this review. The study selection process is illustrated in the PRISMA flowchart (Figure 1).

The following tables provide an overview of the reviewed studies: Table 1 illustrates the variability of physiological foramen morphology in shape, size, and location across different tooth types, anatomical sites, and methodologies. Table 2 outlines the chronological development of imaging techniques in ex vivo research. Table 3 details study authors, country (ISO code), tooth type, sample size (teeth/foramina), as well as measurements of the distance from the physiological foramen to the anatomical apex and foramen diameter. While Table 1 and Table 2 include all 14 studies, Table 3 presents only 10 studies due to missing information in the remaining publications.

## 4. Discussion

This systematic review examined the physiological foramen and associated with this, the variability of the distance between the anatomical apex and the physiological foramen and the diameter of the foramen. The results show a considerable anatomical range that is clinically relevant and must be considered in endodontic treatments to achieve complete root canal filling and avoid over-instrumentation. An individually tailored approach based on tooth and root morphology is therefore crucial. The following discussion classifies the findings in the existing literature and highlights the challenges posed by inconsistent definitions and measurement methods in earlier studies.

### 4.1. Shape of Physiological Foramen

Successful removal of infected tissue and subsequent obturation requires a thorough understanding of the apical morphology [10]. Numerous studies have investigated the shape of the physiological foramen. Different tooth types were examined across studies. While maxillary teeth, particularly molars, show a higher prevalence of oval foramina, especially in the mesiobuccal and palatal root canals (66.67–82.35% oval in Marroquín et al. [7]), Dummer [3] found four types of apical constrictions. Seltzer et al. [24] further demonstrated that inflammatory processes and instrumentation can lead to resorption at the root apex, often resulting in a funnel-shaped foramen appearance, underlining the biological variability and clinical complexity of this region. In incisors, Chapman [16] recorded values between 0.5 mm and 1.25 mm for various maxillary and mandibular specimens. Abarca et al. [13] examined 41 maxillary and 48 mandibular molars and classified the shape of the foramen based on the difference between the smallest and largest diameters: a difference ≤ 0.2 mm was considered round, while a greater difference indicated an oval shape. In the maxillary molars, 50% of the foramina were oval, 32% irregular, and 18% round. In mandibular molars, 59% were oval, 23% irregular, and 18% round [10]. However, definitions varied between studies. Marroquín et al. [7] also reported that the oval shape was most common. In 70% of the maxillary and mandibular molars, an oval apical constriction was observed, followed by the round and then the irregular type. These results were consistent with findings by Abarca et al. [13], Arora & Tewari [14], and Wolf et al. [20,21,22,23]. Chapman studied 120 teeth (20 each of maxillary and mandibular central incisors, lateral incisors, and canines) and found that 100 (83%) exhibited a round apical constriction. Among these, 61 had initially oval canals that became round apically, and 39 were round throughout. In 20 teeth (17%), an oval apical constriction was observed [16]. The clinical relevance of foramen shape lies in the fact that, as Abarca et al. [13] pointed out, endodontic files are designed to create round preparations. Even in the case of non-round foramina, a round preparation should be the goal to enable a uniform obturation up to the physiological foramen [10].

### 4.2. Size of Physiological Foramen

Several studies have reported detailed measurements of foramen diameters, showing a consistent trend: posterior teeth, particularly molars and premolars, tend to have larger foramen diameters than anterior teeth. However, values vary due to differences in methodology, sample size, and anatomical definitions. Chapman [16] found diameters ranging from 0.152–0.174 mm in anterior teeth. Morfis et al. [19] reported average diameters of 0.289 mm in maxillary incisors and 0.263 mm in mandibular incisors, with values increasing to 0.368 mm in mandibular premolars and 0.392 mm in mandibular molars. Marceliano-Alves et al. [17] showed that in maxillary molars, the palatal root diameter increased from 0.34 mm at 1 mm to 0.39 mm at 2 mm from the apex. Mizutani et al. [18] measured diameters in the labiolingual direction and found values of 0.425 mm for central incisors and canines and 0.369 mm for lateral incisors. Olson et al. [25] further demonstrated that in maxillary central incisors, the apical constriction is frequently uneven, challenging the concept of a regular anatomical narrowing. Vertucci [26], in his comprehensive review, also emphasized the high degree of variability in root canal morphology across tooth types, which directly influences instrumentation and obturation strategies. Abarca et al. [10,13] reported diameters of 0.24–0.34 mm in maxillary molars, 0.25–0.33 mm in mandibular molars, and 0.270–0.413 mm in maxillary premolars. Wolf et al. [20,21,22,23] found values of 0.28 mm in mandibular canines and premolars, 0.23 mm in mandibular incisors, and 0.22–0.25 mm in maxillary and mandibular molars. Arora & Tewari [14] found a broader range from 0.174–0.320 mm across molars and premolars. A significant challenge in comparing these findings lies in the diversity of methodologies used to measure the foramen diameter. Differences in sectioning planes, measuring directions (e.g., labiolingual vs. buccolingual), and definitions of the physiological foramen contribute to the wide range of reported values. Future research would benefit from standardized protocols and clear definitions to allow for more consistent and clinically applicable conclusions [27].

### 4.3. Distance Between the Root Apex and the Physiological Foramen

The precise working length in endodontic treatment is critical for ensuring complete root canal obturation while avoiding over-instrumentation. As shown by Ricucci et al. [8] and Wu et al. [9], the physiological foramen should neither be penetrated nor filled beyond its extent, as this can damage periapical tissues and compromise healing. However, the physiological foramen is not readily identifiable on radiographs unlike the anatomical apex, which is commonly used as a clinical reference point. For this reason, several studies have attempted to determine the average distance between the radiographic or anatomical apex and the physiological foramen, with the goal of providing reliable orientation values for clinical practice. Several studies have quantified the average distance between the radiographic or anatomical apex and the physiological foramen to guide clinical practice. Chapman [16] found the foramen 0.5–1.0 mm short of the apex in 92.5% of 120 teeth. Morfis et al. [19] reported distances from the anatomical apex, noting 0.472 mm in maxillary central incisors and 0.977 mm in mandibular incisors, with premolars and molars ranging from 0.418–0.818 mm. Arora & Tewari [14] found wider variation (0.052–2.921 mm) in molars and premolars, and Awawdeh et al. [15] reported a similar range (0.07–2.18 mm). Wolf et al. provided detailed data across root types. In mandibular canines, the distance was 0.45 mm [20]. In maxillary molars, the mesiobuccal, distobuccal, and palatal roots measured 0.82 mm, 0.81 mm, and 1.02 mm, respectively (first molars), and 0.54 mm, 0.43 mm, and 0.63 mm (second molars). In mandibular molars, the mesial and distal roots ranged from 0.78–1.05 mm [23].

### 4.4. Limitations

Several limitations must be considered. First, there is considerable methodological heterogeneity between the studies, particularly regarding lack of clear terminology or nomenclature for the structures examined, which makes it extremely difficult to directly compare the results. Second, there was significant heterogeneity in study design, sample size, population, tooth type, and age, with certain tooth groups being underrepresented. These limitations highlight the need for cautious interpretation of the results and underscore the importance of an individualized approach in clinical endodontics, ideally supported in clinical practice by electronic apex locators and, if necessary, modern imaging techniques.

## 5. Conclusions

This systematic review highlights the critical importance of accurately identifying the physiological apex in endodontics. After analyzing data from 14 studies, the following conclusions can be drawn: in 64% of the studies analyzing the morphology of the physiological foramen, oval-shaped foramina were reported to occur more frequently than round or irregular forms. The findings across studies differ considerably, primarily due to use of different definitions and inconsistent terminology regarding anatomical structures at the root/tooth apex. This variability makes direct comparisons difficult and limits the ability to draw generalized conclusions. Standardized nomenclature and clear definitions of key anatomical landmarks, such as the physiological foramen and anatomical apex, are required. This would enable meaningful comparisons to be made across populations, ages and sexes, and would improve the clinical applicability of research findings.

## Figures and Tables

**Table 1:** Information on study authors, country, methodology, sample size (number and type of foramina examined), foramen shape, and remarks.

Study	Country	Methodology	Sample Size (Number of Foramina)	Oval	Round	Irregular	Remarks
Abarca et al. [10]	CHL	Samples disinfected with sodium hypochlorite, examined under 40× magnification, and photographed.	41 maxillary first molars (97 foramina), 48 mandibular first molars (77 foramina)	50–59%	18%	23–32%	Same criteria for physiological and accessory foramina as above
Abarca et al. [13]	CHL	Teeth sectioned with a diamond saw, placed in sodium hypochlorite for 24 h, and examined under 40× magnification	80 foramina from maxillary first premolars, 89 foramina from maxillary second premolars	72.19%	8.88%	18.93%	Foramina ≥ 0.1 mm considered physiological; <0.1 mm considered accessory. Oval foramina defined as having >0.02 mm difference between the narrowest and widest part
Arora & Tewari [14]	IND	Teeth examined under a stereomicroscope (40× magnification) with software for measurements	100 maxillary first premolars (99 foramina), 100 maxillary second premolars (100 foramina), 100 maxillary first molars (299 foramina), 100 maxillary second molars (293 foramina), 100 mandibular first premolars (100 foramina), etc.	81%	-	-	Foramina ≥ 0.1 mm physiological; <0.1 mm accessory. Oval foramina defined as >0.02 mm difference between narrowest and widest parts
Awawdeh et al. [15]	AUS	Teeth examined under 40× magnification and measured with software	101 mandibular first premolars	50%	23%	27%	Measured major foramen shape
Chapman [16]	GBR	120 teeth stored in saline until examination. Microscopic analysis conducted using Watson microscope (2.7× objective, 10× magnification)	20 maxillary central incisors (30 foramina), 20 maxillary lateral incisors (23 foramina), 20 maxillary canines (26 foramina), 20 mandibular central incisors (27 foramina), 20 mandibular lateral incisors (26 foramina), 20 mandibular canines (28 foramina)	10%	90%	-	Apical constriction defined as the narrowest part of the canal; no exact definition of when a physiological foramen is considered round. Measurement accuracy set at ±0.25 mm
Dummer et al. [3]	GBR	Distance from apex to foramen measured under 20× magnification	23 maxillary central incisors, 22 maxillary lateral incisors, 29 maxillary canines, 38 maxillary premolars, 40 mandibular central incisors, 37 mandibular lateral incisors, etc.	-	-	-	Apical constriction defined as the narrowest part of the canal. No exact number of physiological foramina provided
Marceliano-Alves et al. [17]	BRA	Roots examined radiographically and measured with software	169 foramina from palatal roots of maxillary first molars	-	-	-	Apical constriction defined as the narrowest part of the canal
Marroquín et al. [7]	EGY	Samples cleaned and examined under 40× magnification with software measurements	260 maxillary first molars (780 foramina), 187 maxillary second molars (560 foramina), 76 maxillary third molars (205 foramina), 286 mandibular first molars (556 foramina), etc.	Mesio-buccal 70–82.05%; Palatal 66.47–82.35%	7.33–28.58%	2.44–11.07%	Same criteria for physiological and accessory foramina as above
Mizutani et al. [18]	USA	Roots sectioned in 50-micron steps, stained with cresyl violet, and observed under a stereomicroscope	30 maxillary central incisors (30 foramina), 30 maxillary lateral incisors (30 foramina), 30 maxillary canines (30 foramina)	20% (oval), 23.3% (ovoid)	56.67%	3.3–6.67%	Apical constriction defined as the narrowest part of the canal. No explanation provided for when a foramen is considered round
Morfis et al. [19]	GRC	Apical portion sectioned at 4 mm and examined with Cambridge Stereoscan S150	38 maxillary incisors, 25 mandibular incisors, 29 maxillary second premolars, 92 mandibular premolars, 12 maxillary molars, 17 mandibular molars	-	-	-	No differentiation between central and lateral incisors. Foramina ≥0.1 mm physiological; <0.1 mm accessory
Wolf et al. [20]	CHE/DEU	Teeth treated with chloramine solution, examined via micro-CT, and measured with software	Mandibular canines (122 foramina)	91.1%	7.6%	1.3%	Foramina ≥ 0.2 mm physiological; <0.2 mm accessory. Oval foramina defined as >0.02 mm difference between narrowest and widest parts
Wolf et al. [21]	CHE/DEU	Samples scanned with micro-CT and measured with software	109 mandibular first premolars (139 foramina)	69.1%	16.1%	14.7%	Same criteria for physiological and accessory foramina as above
Wolf et al. [22]	DEU	Teeth treated with hydrogen peroxide and alcohol, examined via micro-CT, and measured with software	125 mandibular incisors (154 foramina)	56%	28.8%	15.2%	Study did not differentiate between central and lateral incisors. Same criteria for physiological and accessory foramina as above
Wolf et al. [23]	DEU	Samples scanned with micro-CT and measured with software	1727 physiological foramina of 516 maxillary and mandibular first and second molars	64.4–86.6%	11.9–25.7%	1.4–14.1%	

**Table 2:** Chronological development of imaging methods in ex vivo research.

	Author	Country	Type of Study	Teeth	Method of Study
1	Chapman [16]	GBR (SCT)	Ex vivo	Permanent teeth	Microscopic Examination (Watson 2.7×)
2	Mizutani et al. [18]	USA	Ex vivo	Permanent teeth	Cresyl fast violet solution/Stereomicroscope
3	Abarca et al. [10]	CHL	Ex vivo	Permanent teeth	Motic Cam (40×)
4	Abarca et al. [13]	CHL	Ex vivo	Permanent teeth	Motic Cam (40×)
5	Arora &Tewari [14]	IND	Ex vivo	Permanent teeth	Magnification (40×)
6	Marroquín et al. [7]	EGY	Ex vivo	Permanent teeth	Magnification (40×)
7	Awawdeh et al. [15]	AUS	Ex vivo	Permanent teeth	Stereomicroscope Magnification (40×)
8	Wolf et al. [20]	CHE/DEU	Ex vivo	Permanent teeth	Micro-CT
9	Wolf et al. [21]	CHE/DEU	Ex vivo	Permanent teeth	Micro-CT
10	Wolf et al. [22]	DEU	Ex vivo	Permanent teeth	Micro-CT
11	Wolf et al. [23]	DEU	Ex vivo	Permanent teeth	Micro-CT
12	Morfis et al. [19]	GRC	Ex vivo	Permanent teeth	Electron Microscope
13	Dummer et al. [3]	GBR (WLS)	Ex vivo	Permanent teeth	Microscope ×20 Magnification
14	Marceliano-Alves et al. [17]	BRA	Ex vivo	Permanent teeth	Micro-CT

**Table 3:** Information on study authors, country (ISO code), tooth type, sample size (teeth/foramina), distance from physiological foramen to anatomical apex, and physiological foramen diameter.

Study	Country	Tooth Type and Number of Teeth/ Foramina	Distance from Apex to Physiological Foramen (mm)	Diameter (mm)
Abarca et al. [10]	CHL	41 maxillary molars (6) with 97 foramina 48 mandibular molars (6) with 77 foramina	-	0.24–0.34 mm (maxillary) 0.25–0.33 mm (mandibular)
Abarca et al. [24]	CHL	80 maxillary premolars (4) with 80 foramina 89 maxillary premolars (5) with 89 foramina	-	0.27–0.413 mm
Arora & Tewari [14]	IND	maxillary first premolars (4) with 99 foramina maxillary second premolars (5) with 100 foramina maxillary first molars (6) with 299 foramina maxillary second molars (7) with 293 foramina mandibular first premolars (4) with 100 foramina mandibular second premolars (5) with 100 foramina mandibular first molars (6) with 200 foramina mandibular second molars (7) with 200 foramina	0.78 mm (max. 4) 0.984 mm (max. 5) MB: 0.996 mm (max. 6) DB: 0.824 mm (max. 6) P: 0.924 mm (max. 6) MB: 0.992 mm (max. 7) DB: 0.632 mm (max. 7) P: 0.719 mm (max. 7) 0.796 mm (mand. 4) 0.781 mm (mand. 5) M: 0.834 mm (mand. 6) D: 0.817 mm (mand. 6) M: 0.78 mm (mand. 7) D: 0.809 mm (mand. 7)	0.171–0.24 mm (max. 4) 0.169–0.254 mm (max. 5) MB: 0. 174- 0.263 mm (max. 6) DB: 0.183–257 mm (max. 6) P: 0.226–0.320 mm (max. 6) MB: 0.168–0.244 mm (max. 7) DB: 0.171–0.230 mm (max. 7) P: 0.218–0.309 (max. 7) 0.173–0.256 mm (mand. 4) 0.158–0.241 mm (mand. 5) M: 0.178–0.261 mm (mand. 6) D: 0.222–0.300 mm (mand. 6) M: 0.198–0.303 mm (mand. 7) D: 0.227–0.323 mm (mand. 7)
Awawdeh et al. [15]	AUS	101 mandibular premolars (4)	0.683 mm	-
Chapman [16]	GB-SCT	20 max. central incisors with 30 foramina 20 max. lateral incisors with 23 foramina 20 max. canines with 26 foramina 20 mand. central incisors & 27 foramina 20 mand. lateral incisors & 26 foramina 20 mand. canines & 28 foramina	In 92.5% of cases, the physiological foramen was located 0.5–1 mm from the apex. In rare cases, it was located 0.25 mm (0.85%), 1.25 mm (5.8%), or 1.5 mm (0.85%) from the apex.	0.174 mm 0.152 mm 0.170 mm 0. 140 mm 0.135 mm 0.157 mm
Dummer et al. [3]	GB-WLS	23 maxillary central incisors 22 maxillary lateral incisors 29 maxillary canines 38 maxillary premolars 40 mandibular central incisors 37 mandibular lateral incisors 28 mandibular canines 54 mandibular premolars	0.85 ± 0.55 mm 0.85 ± 0.55 mm 0.84 ± 0.51 mm 0.95 ± 0.64 mm 0.79 ± 0.55 mm 0.79 ± 0.55 mm 0.95 ± 0.5 mm 0.99 ± 0.57 mm	-
Morfis et al. [19]	GRC	38 maxillary incisors 29 maxillary premolars (5) 12 maxillary molars (6) 25 mandibular incisors 92 mandibular premolars (4 and 5) 17 mandibular molars (6)	0.472 mm 0.816 mm (max. 5) P: 0.429 mm (max. 6) M: 0.665 mm (max. 6) D: 0.418 mm (max. 6) 0.977 mm 0.610 mm (4 and 5) M: 0.531 mm (mand. 6) D: 0.818 mm (mand. 6)	0. 289 mm 0.210 mm (max. 5) P: 0.298 mm (max. 6) M: 0. 235 mm (max. 6) D: 0. 232 mm (max. 6) 0.262 mm 0.368 mm (4 and 5) M: 0.257 mm (mand. 6) D: 0.392 mm (mand. 6)
Marceliano-Alves et al. [17]	BRA	169 foramina of 169 palatal roots of maxillary molars (6)	-	1 mm from apex: 0.34 mm 2 mm from apex: 0.39 mm
Marroquín et al. [7]	EGY	780 maxillary molars (6) 560 maxillary molars (7) 205 maxillary molars (8) 556 mandibular molars (6) 431 mandibular molars (7) 107 mandibular molars (8)	MB: 0.91 mm DB: 0.75 mm P: 0.91 mm Mesial: 0.77 mm Distal: 1.0 mm	MB: 0.24–0.41 mm DB: 0.22–0.33 mm P: 0.33–0.44 mm Mesial: 0.24–0.39 mm Distal: 0.30–0.46 mm
Mizutani et al. [18]	USA	Maxillary central incisors & 30 foramina Maxillary lateral incisors & 30 foramina Maxillary canines with 30 foramina	0.863 mm 0.825 mm 1.010 mm	0.245 mm 0.369 mm 0.375 mm
Wolf et al. [20]	CHE/DEU	122 mandibular canines	0.45 mm (distance from physiological foramen to anatomical apex)	0.28 mm (narrowest part of the physiological foramen); 0.4 mm (widest part of the physiological foramen)
Wolf et al. [21]	CHE/DEU	109 mandibular premolars (4) with 139 foramina	-	0.28 mm (narrowest part of the physiological foramen); 0.37 mm (widest part of the physiological foramen)
Wolf et al. [22]	DEU	125 mandibular incisors with 154 foramina	-	0.23 mm (narrowest part of the physiological foramen); 0.24 mm (widest part of the physiological foramen)
Wolf et al. [23]	DEU	612 maxillary molars (6) 432 maxillary molars (7) 439 mandibular molars (6) 244 mandibular molars (7)	MB: 0.82 mm DB: 0.81 mm P: 1.02 mm Mesial: 0.95 mm Distal: 1.05 mm	MB: 0.24–0.33 mm (narrow/wide) DB: 0.22–0.31 mm (narrow/wide) P: 0.33–0.44 mm (narrow/wide) Mesial: 0.24–0.39 mm (narrow/wide) Distal: 0.30–0.46 mm (narrow/wide)

## Data Availability

The original contributions presented in this study are included in the article/supplementary material. Further inquiries can be directed to the corresponding author.

## Supplementary Materials

The following supporting information can be downloaded at: https://www.mdpi.com/article/10.3390/dj13120581/s1, File S1. PRISMA checklist; File S2. AQUA-Tool (Appraisal tool for QUAlity) assessment; File S3. AQUA-Tool evaluation results.
