# Supplementary material for: Morphology of the Physiological Foramen: A Systematic Review
Source: Dent J (Basel). 2025 Dec 5;13(12):581. doi: 10.3390/dj13120581 (PMC12732147; doi:10.3390/dj13120581)
Supplement: Supplementary file 1 [file dentistry-13-00581-s001.zip › File-S3. AQUA tool evaluation results - need authors confirm-1-resubmitted.pdf]

Supplement - AQUA Tool evaluation

Study	Risk of bias				
	**Target and subject attributed**	**Design of the study**	**Methodology description**	**Descriptive anatomy**	**Reporting of outcomes**
Chapman 1969 [16]	Low	Low	Low	Low	Low
Morfis et al. 1994 [19]	Low	Low	Low	Low	Low
Mizutani et al. 1992 [18]	Low	Low	Low	Low	Low
Dummer et al.1984 [3]	Low	Low	Low	Low	Low
Marılia Marceliano-Alves et al.2016 [17]	Low	Low	Low	Low	Low
Wolf et al. 2017 [23]	Low	Low	Low	Low	Low
Wolf et al. 2020 [21]	Low	Low	Low	Low	Low
Wolf et al. 2020 [22]	Low	Low	Low	Low	Low
Wolf et al. 2021 [20]	Low	Low	Low	Low	Low
Abarca et al. 2014 [10]	Low	Low	Low	Low	Low
Abarca et al. 2018 [13]	Low	Low	Low	Low	Low
Arora et Tewari 2009 [14]	Low	Low	Low	Low	Low
Marroquin et al. 2004 [7]	Low	Low	Low	Low	Low
Awawdeh et al. 2019 [15]	Low	Low	Low	Low	Low
